# Gastrointestinal infection caused by five different strains of *Aeromonas caviae* and one of *Aeromonas veronii*: case report and review of the literature

**DOI:** 10.1186/s12879-026-13287-6

**Published:** 2026-04-14

**Authors:** Gemma Recio Comí, Maria José Figueras, Ana Fernández-Bravo

**Affiliations:** 1 Department of Basic Medical Sciences, Institut d’Investigació Sanitària Pere Virgili (IISPV), Rovira i Virgili University, Institut de Sostenibilitat Canvi Climàtic i Transició Energètica (IURESCAT), Reus, Spain; 2 Laboratori Clínic Camp de Tarragona-Terres de l’Ebre, Hospital de Tortosa Verge de la Cinta, Institut Català de la Salut, Tortosa, Spain; 3 Institut d’Investigació Sanitària Pere Virgili (IISPV), Tortosa, Spain

**Keywords:** *Aeromonas caviae*, *Aeromonas veronii*, Gastroenteritis, Immunosuppressed patient, Mixed infection, Polymicrobial infection, MALDI-TOF MS, ERIC-PCR, *rpoD* gene, Case report

## Abstract

**Background:**

*Aeromonas* species are considered opportunistic emerging pathogens with diarrheal disease being the most common manifestation. Infections involving more than one strain or species of *Aeromonas* are rarely documented, and their impact in the development of the infection and in the treatment has not been thoroughly investigated.

**Case presentation:**

We report a case of a 33-year-old woman with a history of an autoimmune disease, who presented at the emergency department complaining of abdominal pain, fever, acute diarrhea and vomiting. The patient was diagnosed with gastroenteritis caused by *Aeromonas caviae*, based on the identification of the bacteria from a single isolate using the matrix-assisted laser desorption ionization time of flight mass spectrometry (MALDI-TOF MS). Further investigations, from a new culture, after genotyping eight isolates using ERIC-PCR and identification based on *rpoD* gene sequences, revealed the presence of two different *Aeromonas* species and six distinct strains, five belonging to *A. caviae* and one to *Aeromonas veronii.* A review of published *Aeromonas* mixed infection cases was performed to determine the current knowledge.

**Conclusions:**

Standard diagnostics rely on the identification and evaluation of the antimicrobial susceptibility of a single isolate taken from the culture media. This case aims to raise awareness among clinicians of the existence of polymicrobial infections and highlights the limitations of isolating a single colony. This may underestimate the genetic diversity within a sample, as different clones of the same or different species with varying antimicrobial resistance can potentially impact treatment response and the evolution of the infection. The reviewed cases of polymicrobial *Aeromonas* infections highlight the limited knowledge regarding their true incidence and potential clinical impact, which may influence antibiotic treatment and patient outcomes and underscores the need for further research.

**Supplementary Information:**

The online version contains supplementary material available at 10.1186/s12879-026-13287-6.

## Background

 The genus *Aeromonas* has expanded considerably and at present includes thirty-eight knownspecies of which only thirty-four have been validly published. At least eighteen of those have been recovered and associated with human infections but four species *Aeromonas caviae*, *Aeromonas veronii*, *Aeromonas dhakensis*, and *Aeromonas hydrophila* account for 95.3% of the recovered isolates when housekeeping genes are used for the identification [1, 2]. The most frequent clinical presentations ordered by their prevalence are diarrhea, wound infections and bacteremia [1, 3]. *Aeromonas* gastroenteritis is generally a self-limiting process consisting of diarrhea, abdominal pain, fever, nausea and vomiting, acquired mainly through ingestion of contaminated water and food [4–7].

Co-isolation of two distinct *Aeromonas* strains or species in the same clinical sample has been seldomly reported and mainly in association with wound or soft tissue infections [8–18] and more rarely in patients with gastroenteritis [19, 20]. Mosser et al. (2015) [13] have estimated that globally *Aeromonas* polymicrobial infections may occur with a minimal frequency of 5% despite in the study performed by Lamy et al. (2009) [10] the incidence recorded was 9%.

This study aims to describe a case of diarrhea caused by *Aeromonas* in an immunocompromised patient from whom five different strains of *A. caviae* and one strain of *A. veronii* were isolated from the same fecal sample using genetic identification methods, and to review previously reported cases in the literature. The antimicrobial susceptibility pattern of the individual strains and their behavior in combination with the other strains was also investigated.

## Case presentation

A 33-year-old woman presented to the emergency department of the University Hospital Joan XXIII in Tarragona (Spain) with one-week history of abdominal pain, diarrhea and vomiting, accompanied by fever of 37.7 °C for the last 24 h. The patient had a history of systemic scleroderma, an autoimmune connective tissue disease affecting skin and internal organs [21], and has been on cyclosporine therapy, an immunosuppressant medication, for the last 10 years. Initial laboratory blood tests revealed neutrophilic leukocytosis with a white blood cell count of 14.6 × 10^9^/L (normal range: 4–12.9 × 10^9^/L), a neutrophil count of 13.6 × 10^9^/L (normal range: 2–7 × 10^9^/L) and an elevated C-reactive protein (CRP) level of 1.5 mg/dL (normal range: <1 mg/dL). With all this data, the patient was diagnosed with a probable case of gastroenteritis. No aquatic exposure, trauma, or specific food consumption was recorded in clinical history. Intravenous fluid was initiated in the emergency department, together with an empirical intravenous treatment with ciprofloxacin 400 mg/200 mL every twelve hours, for seven days. Given the patient’s underlying autoimmune disease, immunosuppressed status, and one-week history of symptoms, she was admitted to the Internal Medicine Department for further evaluation and management. A stool sample was collected and cultured for the presence of bacterial enteropathogens i.e., *Campylobacter* spp. (Campylosel Agar, bioMérieux^®^, Marcy-l’Étoile, France), enterohemorrhagic *Escherichia coli* (EHEC) serotype O157:H7 (MacConkey Agar with sorbitol, bioMérieux^®^), *Salmonella* spp. and *Shigella* spp. (Salmonella-Shigella Agar, bioMérieux^®^), *Yersinia* spp. and *Aeromonas* spp. (*Yersinia* Selective Agar (Cefsulodin-Irgasan-Novobiocin Agar (CIN)), bioMérieux^®^). Also, a screening of *Clostridioides difficile* (= *Clostridium difficile*) was performed for the detection of glutamate dehydrogenase (GDH) through enzyme immunoassay (CerTest *Clostridium difficile* antigen GDH, CerTest Biotec^®^, Zaragoza, Spain).

Only the stool culture on CIN was positive after 24 h incubation at 37 °C. The identification of the bacteria using a single colony was performed with the matrix-assisted laser desorption/ionization time of flight mass spectrometry (MALDI-TOF MS, Bruker Daltonics GmbH, Bremen, Germany) that uses the Biotyper Real Time Classification software v3.1 including database V8. The bacteria initially identified based on a single colony correspond to *Aeromonas caviae*, with a score of 2.540, that indicated a highly probably species identification [22].

The antibiotic susceptibility from the single isolate mentioned before was performed with MicroScan Walkaway 96 plus ID/Antimicrobial susceptibility testing (AST) system (Beckman Coulter, Inc., Brea, California, USA) and the Minimum Inhibitory Concentration (MIC) values were analyzed according to the European Committee on Antimicrobial Susceptibility Testing (EUCAST) v 16.0 breakpoint tables (publicly available at the www.eucast.org website, accessed March 2026) and the 2016 3rd Edition of Clinical and Laboratory Standards Institute (CLSI) M45 guideline [23], revealing that the single initially studied isolate showed only to be resistant to ampicillin and susceptible to the other antimicrobial tested. So, the laboratory informed the clinician of a case of diarrhea produced by *A. caviae* resistant to ampicillin. Although the patient saw pronounced improvement of the diarrhea after a few days of antibiotic treatment, complications during hospitalization appeared, i.e., a chronic anemia flare-up and a pleural effusion consequence of intravenous fluid therapy and she remained hospitalized for ten days before she could be discharged.

**Fig. 1:**
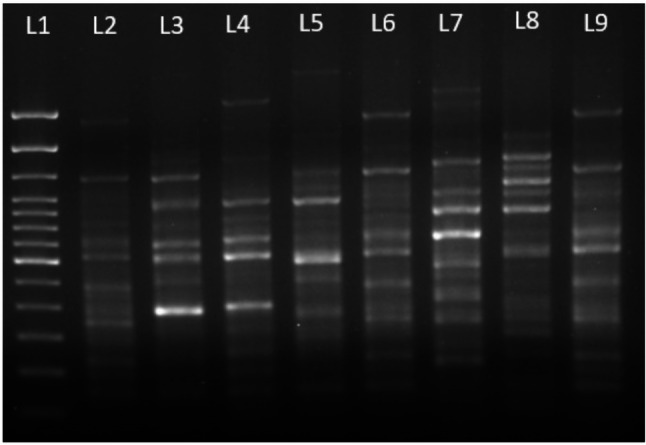
ERIC-PCR profile of the eight *Aeromonas* isolates showing six different patterns. Lane L1, Molecular weight ladder; Lanes L2-L9: Isolate 1; Isolate 2; Isolate 3; Isolate 4; Isolate 5; Isolate 6; Isolate 7 and Isolate 8. Notice that isolates 1, 5 and 8 showed the same pattern

**Fig. 2:**
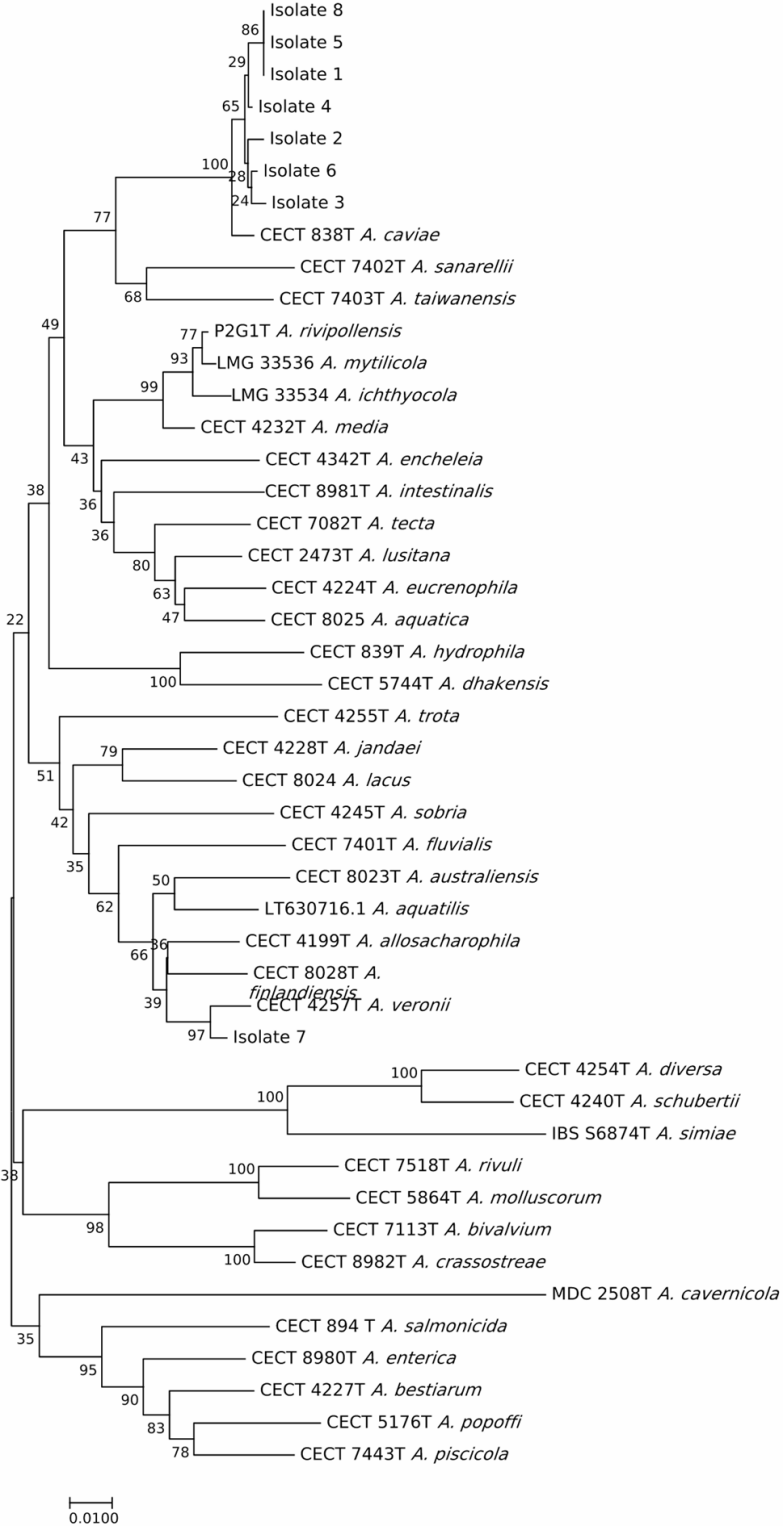
Phylogenetic tree of the *rpoD* sequences of the 8 isolates and the *Aeromonas* spp. type strains. Notice that all isolates cluster with the sequence of the type strain of *A. caviae* with the only exception of isolate 7 that clusters with the type strain of *A. veronii*

This case was integrated into a broader research study aiming at determining the prevalence of different strains and/or species of *Aeromonas* associated to cases of diarrhea using a specific protocol. The latter consisted of culturing again the positive fecal samples on CIN Agar using the quadrant streaking method and, after 24 h of growth, eight separate colonies, mainly from the third and fourth quadrants were picked up to perform a new MALDI-TOF MS identification (Table 1). This was done in order to maximize the probability to ensure diversity and to increase the likelihood of sampling well-isolated and distinct colonies. In addition, these isolates were genotyped using the Enterobacterial Repetitive Intergenic Consensus Polymerase Chain Reaction (ERIC-PCR). This is a DNA fingerprinting technique that amplifies short and repetitive sequences of the bacterial genome, generating strain-specific banding patterns after electrophoresis, using the primers and conditions described by Versalovic et al. [24] (Fig. 1; Table 1). Thereafter, the isolates were genetically identified by sequencing the *rpoD* gene using the primers and conditions described by Soler et al. [25] (Table 1). The sequences of the type strains of the 38 described *Aeromonas* species were aligned using the ClustalW algorithm, and the phylogenetic analysis was performed with the Neighbour-Joining (NJ) algorithm in MEGA v 6.0 [1] (Fig. 2).

**Table 1:** Results of the ERIC-PCR genotyping, the identification with MALDI-TOF MS and *rpoD* gene phylogeny

Isolate	ERIC Genotype	MALDI-TOF MS Identification		Species with *rpoD*
		Species	Score	
1	G1	*A. caviae*	2.189	*A. caviae*
2	G2	*A. caviae*	2.322	*A. caviae*
3	G3	*A. caviae*	2.141	*A. caviae*
4	G4	*A. caviae*	2.111	*A. caviae*
5	G1	*A. caviae*	2.292	*A. caviae*
6	G5	*A. caviae*	2.061	*A. caviae*
7	G6	A. caviae*	2.134	*A. veronii*
8	G1	*A. caviae*	2.240	*A. caviae*

Scores ≥ 2.300 indicate highly probable species identification and ≥ 2.000–2.299 only probable [22]. *Initially misidentified as *A. caviae* by MALDI-TOF MS and identified correctly as *A. veronii* based on the phylogenetic analysis of the sequences of the *rpoD* gene as show in Fig. 2

Moreover, the antimicrobial susceptibility phenotype of the different strains identified on the basis of ERIC-PCR was determined, using the MicroScan AST system (Beckman Coulter, Inc.) both individually and in mixed combinations. MIC interpretations were performed according to EUCAST (tables publicly available at the www.eucast.org website, accessed March 2026). For antibiotics without EUCAST breakpoints, CLSI criteria were applied [23]. For antibiotics lacking breakpoints in both guidelines, interpretations were based on *Enterobacteriaceae* breakpoints of EUCAST guidelines commented. In addition, all isolates exhibited an identical phenotype in the biochemical tests included in the MicroScan ID/AST panel (acid production from glucose, sucrose, and arabinose, indole production, β-galactosidase activity (ONPG test), nitrate reduction, and oxidative/fermentative (O/F) glucose metabolism) and were all identified as belonging to the *Aeromonas caviae* complex.

## Discussion and conclusion

The genus *Aeromonas* comprises Gram-negative microorganisms that are ubiquitous in aquatic environments, including surface water, groundwater, drinking water, bottled water, wastewater, seawater and irrigation systems. These environments can act as sources of contamination for food products such as fruits, vegetables, dairy products, meat, fish and shellfish [3, 5, 26]. Consumption of contaminated water or food is considered the main route of gastrointestinal infection caused by *Aeromonas* [3, 26, 27]. However, no clear exposure history was not recorded in the patient described in this study.

According to the literature, *Aeromonas* gastroenteritis is relatively frequent among patients with comorbidities and/or immunosuppression [3, 20, 28–31], with a reported incidence ranging from ~ 13% to 80% across cohorts [7, 10, 32, 33]. In the studies of Lamy et al. [10] and Sagas et al. [33], patients with underlying morbidities accounted respectively for 60% and 44.3%, of the total cases. Higher values were reported in a study conducted in Spain, where up to 77% of patients had comorbidities and 26.9% were receiving immunosuppressive therapy [7]. Obi et al. [32] detected *Aeromonas* in 13.3% of HIV-infected patients with chronic diarrhea in South Africa. The patient presented here, a woman of around thirty years, was receiving immunosuppressive therapy, which likely heightened her susceptibility to severe infection. These observations reinforce the role of *Aeromonas* as an opportunistic enteropathogen in immunocompromised individuals. Despite this, no European clinical guidelines specifically address the treatment of diarrhea caused by *Aeromonas*. However, the Infectious Diseases Society of America (IDSA) guidelines recommend considering antimicrobial therapy in immunocompromised patients with persistent diarrhea when a bacterial pathogen such as *Aeromonas* is isolated [34] particularly in moderate or severe cases. In our case, ciprofloxacin treatment resulted in complete symptom resolution within a few days.

The clustering observed in the phylogenetic analysis based on the *rpoD* gene supported that five strains belonged to *A. caviae* and one to *A. veronii*, showing > 98% similarity to the sequence of their corresponding type strains (Fig. 2). However, MALDI-TOF MS had identified all strains as belonging to a single species (*A. caviae*) (Table 1). Misidentifications of *Aeromonas* spp. using MALDI-TOF MS have been documented in many studies [22, 35–37] and misidentification of *A. veronii* varied between 100% and 32.2% using the Biotyper Real-Time Classification software of Bruker Daltonics as reported in a recent review [22]. MALDI-TOF MS is a rapid and widely used system in the routine microbiology laboratory workflow, but many laboratories are not aware about the limitations of the method. The limited number of strains and species included in the MALDI-TOF MS database is the factor that hamper the correct identification, and a proper update should be the responsibility of the company commercializing the system [22]. When molecular identification methods are applied the two species identified in this study, *A. caviae* and *A. veronii*, are among the most frequently reported species in association with human infections especially in association with gastroenteritis representing 64.6% and 53.1%, respectively of the feces isolates [1]. In studies of adults diarrhea due to *Aeromonas* from different countries *A. caviae* was identified with an incidence of 43.9% in China, 65% in Israel and 75% in Italy [38–40]. Conversely, *A. veronii* was the predominant species in Taiwanese adults (52.6%) and in an Australian cohort [41, 42].

Few studies have reported polymicrobial infections associated with two *Aeromonas* species or strains, as shown in Table S1, and most of them corresponded to wound infections. However, many of the identifications were done with phenotypic biochemical tests, which have limited discriminatory power and most of them tend to incorrectly identify the species as belonging to *A. hydrophila* [43–45]. Our case report is noteworthy because, to the best of our knowledge and based on the literature reviewed (Table S1), it represents the first reported case of diarrhea due to a mix of five different strains of *A. caviae* and one of *A. veronii* recognized after genotyping and species identification using the sequences of the *rpoD* gene, a recognized reference molecular methods for *Aeromonas* identification and genotyping [46].

Specific screening for the presence of *Aeromonas* in cases of diarrhea is not routinely performed in many clinical laboratories, and its roles as enteropathogen is still underestimated by some clinicians [47]. According to Janda and Abbott [3], isolation of *Aeromonas* from stool samples is relatively simple as these bacteria can grow on most enteric isolation media. However, CIN Agar, used in clinical laboratories for the isolation of *Yersinia* spp., can be considered a multifunctional and cost-effective approach that allows the growth of *Aeromonas* spp., showing the typical bull’s eye-like colony due to fermentation of D-mannitol [3]. This morphology facilitates presumptive identification and can help prevent *Aeromonas* from being overlooked when using standard enteric isolation media. In fact, this medium has been widely used for the recovery of *Aeromonas* from stool samples [22, 48–50] and was the medium from which we identified a mixed infection of *Yersinia enterocolitica* and *A. caviae* [49].

Two antimicrobial susceptibility patterns were identified among the six strains analyzed. Antibiotype 1, that corresponded to the typical intrinsic resistance to ampicillin observed in all *Aeromonas* species, with the exception of *Aeromonas trota* [4] and antibiotype 2, that showed additionally resistance to nalidixic acid and susceptibility to the latter with increased exposure to ertapenem. Resistance to nalidixic acid in *A. caviae* and *A. veronii* has been associated with mutations in the quinolone resistance-determining regions (QRDRs) of the *gyrA* and *parC* genes [51, 52]. Despite that, the fluoroquinolone ciprofloxacin is used as a first-line antibiotic to treat *Aeromonas* infections [51, 53]. However, treating with this drug infections caused by these bacteria already carrying a first mutation in the mentioned genes can promote the development of other new mutations, increasing the risk of selecting fluoroquinolone-resistant mutants [54, 55] that maybe responsible of the treatment failure [48, 56, 57]. In the same way, resistance to ertapenem has been described [58, 59] to be frequently mediated by the *cphA* gene, which encodes the metallo-β-lactamase CphA, with activity against carbapenems [51]. The presence of carbapenemase-producing-*Aeromonas* is of public health concern since carbapenems are considered the last-resort drug to be used to treat serious, multi-drug resistant infections when other antimicrobials have failed [60].

It is also worth noting that we found strains sharing the same antibiotype that belonged to a different ERIC-PCR genotype and species (strains 1–3 of *A. caviae* (G1-G3) and strain 6 of *A. veronii* (G6) showed antibiotype 1; strains 4 (G4) and 5 (G5) of *A. caviae* showed antibiotype 2), demonstrating that the antimicrobial susceptibility patterns don’t seem to be adequate for typing purposes as indicated in previous studies [61–63] and as by the European Society of Clinical Microbiology and Infectious Diseases (ESCMID) guidelines in 2007 [64]. The co-existence of more than one strain of *Aeromonas* of the same species has been recognized in some studies, thanks to the different antimicrobial resistance patterns observed for the isolated strains [Table S1, 11, 15]. Furthermore, genetically identical isolates may still display different susceptibility profiles due to differential expression of resistance determinants, as illustrated by the overexpression of the *ImiS* metallo-β-lactamase in a case of *A. veronii* cholangitis [59].

Another interesting fact is that the antimicrobial susceptibility pattern informed in our study for the original single isolate investigated at the hospital clinical laboratory corresponded to antibiotype 1. We could assume that the isolate selected probably corresponded to strains 1–3 or 6 as subsequently identified. This implies that strains 4 and 5 [which were resistant to nalidixic acid and showed an intermediate susceptibility to ertapenem (= SIE, susceptibility increased exposure)] were not represented in the antibiogram report given to clinicians to advise on patient therapy. Despite this, no impact on treatment response or on the course of the infection was observed in our patient who exhibited a good response to ciprofloxacin in a few days. However, as explained above, treating with fluoroquinolones strains that may present a first mutation in the quinolone target genes may result in treatment failure and lead to the selection and spread of resistant mutants [55, 57]. If, in our patient, the infection had evolved into a bacteremia (i.e., septicemia) in which carbapenem could have been the treatment of choice, the patient could have received an inadequate dosing regimen guided by the report indicating carbapenem susceptibility, without information of the SIE that could potentially lead to a treatment failure.

This study presents a very unusual case, partly due to its true rarity and partly because its detection is for sure underestimated, because the diagnosis, as commented, is routinely made in the clinical laboratory based on the characterization of a single colony taken from the culture media [8, 65–67], as occurred in our case. Polymicrobial infections are increasingly recognized as important contributors to disease severity, due to interactions between co-existing microorganisms that may enhance virulence or alter antimicrobial responses [67–69]. However, this aspect is rarely considered in routine diagnostic workflows, which typically rely on the analysis of a single colony from culture plates. Our review of experimental studies (Table S2) suggests that synergistic interactions between co-isolated *Aeromonas* strains may increase virulence, highlighting the need for further research.

Although the mixed cultures in our case did not seem to increase the resistance to antibiotics in comparison with the single strains, several studies have demonstrated that interactions within microbial communities may modulate the response to antimicrobials, either increasing tolerance [70, 71] or enhancing susceptibility and their efficacy [72]. In this sense, Fernández-Bravo et al. [49] recently performed the first study investigating the interaction between *A. caviae* with *Yersinia enterocolitica* in a diarrheal case, demonstrating that co-infection increased the resistance to the tested antibiotics. Since antibiotic choice and dosage are based on the AST of a single isolate, clinicians should be alerted that this could not represent the reality of some polymicrobial infections. Given that approximately 50% of *Aeromonas*-associated gastroenteritis cases required antibiotic therapy in the studies reviewed [29, 31, 33, 41, 48, 73], the involvement of multiple strains or species with potentially different susceptibility profiles may have important implications in prognosis and treatment. In cases of poor clinical progression, particularly in young children (< 5 years) or elderly patients with comorbidities, it may be advisable to repeat microbiological testing, either by reanalyzing the original specimens or collecting new samples. This will allow to identify additional strains or species potentially involved in the infection and determine their individual AST profiles in order to enable a more accurate diagnosis and tailored treatment.

In conclusion, this study highlights that polymicrobial infections involving multiple *Aeromonas* strains or species may be under-recognized in routine clinical practice. Conventional diagnostic approaches based on a single isolate may not fully reflect the microbial diversity present in clinical samples, potentially affecting treatment decisions. Improved awareness of this phenomenon may contribute to better diagnosis, antimicrobial stewardship, and understanding of the clinical impact of polymicrobial infections.

## Supplementary Information

Below is the link to the electronic supplementary material.

Supplementary Material 1

Supplementary Material 2

Supplementary Material 3

## Data Availability

All data generated or analyzed in this study are included in this article. The sequences of the *rpoD* gene have been deposited in GenBank under the accession ID 3046984.

## Abbreviations

Act: Cytotonic enterotoxin
AST: Antimicrobial susceptibility testing
CIN: Cefsulodin Irgasan Novobiocin
CLSI: Clinical and Laboratory Standards Institute
CRP: C-reactive protein
ERIC-PCR: Enterobacterial Repetitive Intergenic Consensus-PCR
ESCMID: European Society of Clinical Microbiology and Infectious Diseases
EUCAST: European Committee on Antimicrobial Susceptibility Testing
ExoA: Exotoxin A
GDH: Glutamate dehydrogenase
IDSA: Infectious Diseases Society of America
MALDI-TOF MS: Matrix-assisted laser desorption/ionization time of flight mass spectrometry
MIC: Minimum Inhibitory Concentration
QRDR: Quinolone resistance-determining region
T3SS: Type 3 secretion system
T6SS: Type 6 secretion system
WGS: Whole genome sequencing
YER: Yersinia Selective Agar
