# Supplementary figures and images for: Gastrointestinal infection caused by five different strains of Aeromonas caviae and one of Aeromonas veronii: case report and review of the literature
Source: BMC Infect Dis. 2026 Apr 14;26:1016. doi: 10.1186/s12879-026-13287-6 (PMC13202911; doi:10.1186/s12879-026-13287-6)

labmicro 2018-10-19 10hr 53min

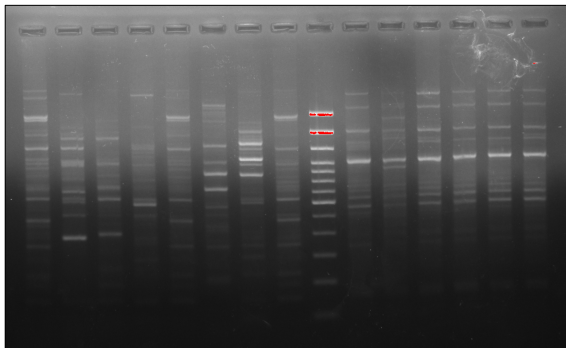

Supplement: Supplementary file 1 — Supplementary Material 1 [file 12879_2026_13287_MOESM1_ESM.pdf]
