# Supplementary material for: Gastrointestinal infection caused by five different strains of Aeromonas caviae and one of Aeromonas veronii: case report and review of the literature
Source: BMC Infect Dis. 2026 Apr 14;26:1016. doi: 10.1186/s12879-026-13287-6 (PMC13202911; doi:10.1186/s12879-026-13287-6)
Supplement: Supplementary file 2 — Supplementary Material 2 [file 12879_2026_13287_MOESM2_ESM.docx]

Table S1. Cases involving the isolation of different *Aeromonas* strains or species in the same clinical sample

| ***Aeromonas* species (Nº of strains per patient or sample)** | **Origin** | **ID method** | **Comments** | **Country/ Reference** |
| --- | --- | --- | --- | --- |
| *A. sobria* (1)  *A. hydrophila* (1) | Aspirate obtained from a non-suppurative 10-mm-deep leg puncture wound sustained during diving in low-salinity water in a 19yo male diver patient presenting with decompression sickness | Conventional phenotypic tests and API20E | Both species were recovered in the initial culture, but only *A. hydrophila* was isolated in a culture performed 3 days later. The patient received hyperbaric therapy and 10 days of tetracycline, with clinical improvement observed after 3 days. The *A. sobria* strain produced fluid accumulation in the rabbit ligated loop assay but did not exhibit a cholera enterotoxin–like effect present in the tetracycline-resistant *A. hydrophila* strain. The origin of the infection was associated with exposure of the diver’s wound to water, as an ongoing parallel survey conducted at the same diver’s training site, where cultures were performed from the ears and face masks of divers after a 30-min exposure revealed bacterial types similar to those present in the river (Seidler et al., 1980).  The species referred as *A. sobria* corresponds to *Aeromonas veronii* biotype *sobria* according to updated taxonomic criteria (Joseph et al., 1991). | USA/ Joseph et al., 1979 [8] |
| *A. hydrophila* (1)  *A. caviae* (1) | Stool from a 49yo female with epigastric pain, vomiting and watery diarrhoea | Conventional phenotypic tests and API20E | Several distinct colony types were observed in blood agar, 2 of them being positive to cytochrome oxidase and showing two distinct API20E phenotypic profiles. Additional phenotypic characterization confirmed their identity as 2 different species. | USA/ Janda et al., 1984 [90] |
| *A. sobria* (1)^a^  *A. caviae* (1) | Stool from a 20mo male with bloody diarrhoea | Conventional biochemical and serological methods | In a two-year study to determine the clinical significance of *Aeromonas* in cases of diarrhoea, 214 patients were examined. Among them, 10 had two *Aeromonas* species detected in their stool samples, but data were available for only 6 patients. Authors identified two significant risk factors for diarrhoea: consumption of untreated drinking water and a prior antibiotic therapy. Notably, five patients developed diarrhoea after treatment with penicillin or ampicillin for an otitis media and another after been treated for a urinary tract infection. | USA/ Moyer et al., 1987 [19] |
| *A. hydrophila* (1)  *A. caviae* (1) | Stool of 5 patients; 3 males: 2mo; 49yo and 21yo and 2 females: 70yo and 25yo |  |  |  |

^a^The species referred as *A. sobria* corresponds to *Aeromonas veronii* biogroup *sobria* according to updated taxonomic criteria (Joseph et al., 1991)

Table S1. Cases involving the isolation of different *Aeromonas* strains or species in the same clinical sample. Continued 1

| ***Aeromonas* species (Nº of strains per patient or sample)** | **Origin** | **ID method** | **Comments** | **Country/ Reference** |
| --- | --- | --- | --- | --- |
| *A. hydrophila* (2) | Stool from a 16mo male | *Aeromonas* specific phenotypic tests | The authors investigated whether patients with *Aeromonas*-associated diarrhoea were simultaneously colonized by multiple intestinal strains using a genotyping method (whole-cell DNA restriction endonuclease analysis, REA) and found that two of the 11 patients who had a second or third additional culture performed showed more than one isolate of *Aeromonas.* One 16mo patient developed *A. hydrophila-*associated diarrhoea while being treated with amoxicillin for otitis and after a change to cotrimoxazole the diarrhoea ceased in a week. A second stool (day 10) was negative, but a third sample (day 21) showed two different *A. hydrophila* strains. The other, 1yo patient, presented gastroenteritis and *Salmonella typhimurium* and *A. veronii* were isolated from the same fecal sample*.*  The same *A. veronii* strain persisted on day 4 but was replaced by a different isolate by day 14. As indicated by the authors the findings suggest that, during diarrhoea, the intestine may be colonized by multiple *Aeromonas* isolates. | Netherland/Kuijper et al., 1989 [91] |
| *A. veronii* (2) | Stool from a 1yo female |  |  |  |
| *A. veronii* (1)  *A. jandaei* (1) | Purulent lacrimal sac–cutaneous fistula (wound) in a 10yo male patient | Phenotypic tests and API20E/ DNA-DNA hybridization (DDH) | A mixed infection with two different *Aeromonas* spp. is reported. Five weeks before admission, the patient suffered an apparently superficial air-rifle injury in the left lacrimal area. Two days later, after swimming in a freshwater pond, erythema and mucopurulent drainage developed, and the patient went to the emergency department, where a sample was collected and a treatment with cephalothin was initiated. Persistent symptoms prompted radiological examination, which revealed a metallic foreign body in the lacrimal sac, leading to the excision of a subcutaneous lacrimal fistula. The initial culture contained two isolates, one resembling *A.hydrophila* and the other *Vibrio cholerae,* but the DDH revealed that they belonged respectively to *A. veronii* and *A. jandaei.* The latter showed the characteristic cephalothin resistance of this species, while the former species was susceptible. The antibiograms performed explained the failure of cephalothin therapy, which was then changed to cefotaxime and trimethoprim-sulfamethoxazole. The source of infection was the exposure of the wound  to a contaminated freshwater pond. A caution was raised regarding the potential | USA/ Joseph et al., 1991 [9] |

Table S1. Cases involving the isolation of different *Aeromonas* strains or species in the same clinical sample. Continued 2

| ***Aeromonas* species (Nº of strains per patient or sample)** | **Origin** | **ID method** | **Comments** | **Country/ Reference** |
| --- | --- | --- | --- | --- |
|  |  |  | synergistic role of multiple *Aeromonas* species in human pathogenesis, which were considered to be probably more common than recognized. |  |
| *A. hydrophila* (1)  *A. caviae* (1) | Stool from a 62yo patient | Phenotypic tests and API20E | The authors studied the prevalence of *Aeromonas* spp. isolated from clinical and freshwater samples (i.e., rivers and artificial basins that supply raw water to drinking water treatment plants) in Southern Italy. Two different *Aeromonas* species were recovered from two stool samples of two patient, one presenting with diarrhoea, vomiting, abdominal pain, and fever and the other with NS symptoms but strains showed multiple antibiotic resistances as did the *Aeromonas* isolated from the water raising concerns about their potential threat to public health. | Italy/ Dumontet et al., 2003[20] |
| *A. veronii* (1)  *A. caviae* (1) | Stool sample, further data NS |  |  |  |
| Neither the species nor the Nº of strains was specified. | Mainly from wounds and soft tissue infections (44% of the total cases) | *rpoB* gene sequencing | A prospective six-month multicentre, nationwide French study of 78 cases of *Aeromonas* infections among which 7 (9%) patients showed more than one strain mainly in wound infections. The study alerted that such cases of co-infection may be underrecognized, because mixed cultures are not systematically investigated. | France/ Lamy et al., 2009 [10] |
| *A. hydrophila* (2) | Stump tissue cultures from successive analysis after limb amputation due to a necrotizing fasciitis (NF) | Aerokey II (phenotypicbiochemica l tests) and genomic sequencing | The NF evolved from a wound of a young immunocompetent girl who fell into a river (while zapping). Of the four isolates (NF1–NF4) recovered, NF2–NF4 were clonal, while NF1 (the initial wound isolate) was genetically distinct. Further studies were done with those strains to study their virulence and the synergetic or antagonistic effects using cell cultures and animal models (Ponnusamy et al., 2016; Fernández- Bravo et al., 2016) and are described in Table 4. | USA/ Grim et al., 2014 [12] |

Table S1. Cases involving the isolation of different *Aeromonas* strains or species in the same clinical sample. Continued 3

| ***Aeromonas* species (Nº of strains per patient or sample)** | **Origin** | **ID method** | **Comments** | **Country/ Reference** |
| --- | --- | --- | --- | --- |
| *A. media* ^b^ (1)  *A. veronii* (1) | Stool of a 5mo child that showed fever and predisposing comorbidity factors^c^ | *gyrB* and *rpoB* sequencing | The 6 pairs of strains listed were recovered from 2 Spanish and 4 French patient~~s~~ with *Aeromonas* mixed infections from diverse origins that were used to investigate potential synergies and interactions using the *Caenorhabditis elegans* virulence model (results are described in Table 4). Information on the 5mo stool sample and 62yo respiratory tract sample comes from Robert et al. (2023) study, in which they developed a *Drosophila melanogaster* systemic infection model, enabling the discrimination of the specific effects of bacterial mixtures in the modulation of the virulence (Table 4). | France/ Mosser et al., 2015 [13] |
| *A. hydrophila* (1)  *A. veronii* (1) | Respiratory tract sample from a 62yo man with comorbidity factors^d^ |  |  |  |
| *A. veronii* (1)  *A. hydrophila* (1) | Wound, further data not specified (NS) |  |  |  |
| *A. media* *(*1)  *A.veronii* (1) | Respiratory tract, further data NS |  |  |  |
| *A. sanarelli* (1)  *A. veronii* (1) | Wound, further data NS |  |  |  |
| *A. caviae* (2) | Stool, further data NS |  |  |  |

^b^The strain of *Aeromonas media* was later reclassified by Talagrand-Reboul et al. (2017) as *Aeromonas rivipollensis* (Marti & Balcázar, 2015); ^c^Hydrocephalus with a ventriculoperitoneal shunt, and a catheter-associated coinfection; ^d^Bronchiectasis undergoing corticosteroid therapy; NS: not specified

Table S1. Cases involving the isolation of different *Aeromonas* strains or species in the same clinical sample. Continued 4

| ***Aeromonas* species (Nº of strains per patient or sample)** | **Origin** | **ID method** | **Comments** | **Country/ Reference** |
| --- | --- | --- | --- | --- |
| Two different *Aeromonas* spp. (NS) were found in two patients | The origin of isolation was NS but 83% of the studied strains came from skin and soft tissue infections | API 20NE, MicroScan WalkAway and Vitek 2 biochemical systems | The study analysed a 13-year database (2000–2013) from public laboratories in Australia’s Northern Territory for VACS (*Vibrio* spp., *Aeromonas* spp., *Chromobacterium violaceum*, and *Shewanella* spp.) to compare their relative frequencies, characteristics, and antimicrobial susceptibilities. | Australia/ McAuliffe et al., 2015 [14] |
| *A. veronii* (2) | Cultures from an infected breast reconstruction flap, obtained two days after leech therapy for venous congestion | MALDI-TOF and whole genome sequencing (WGS) | After mastectomy and flap reconstruction, leech therapy for venous congestion was applied from days 2 to 7. On day 3, the patient was febrile, and the infection progressed to necrotizing cellulitis despite several surgeries and antibiotics. Cultures (day 14) yield two distinct isolates with different antimicrobial susceptibility profiles: one multidrug-resistant, producing an extended-spectrum β-lactamase (ESBL) and resistant to fluoroquinolones and aminoglycosides, and another resistant only to fluoroquinolones. Targeted therapy was initiated, and the patient was discharged on day 28 with cefepime that was continued until day 50. The infection was associated to leech therapy, as leeches are known to possess *Aeromonas* as symbionts to digest blood (Graf, 2015). | France/ Barraud et al., 2020 [15] |
| *A. encheleia* (1)  *A. bestiarum* (1) | A knee swab from an adult male patient with bursitis yielded two species isolated from the same sample | MALDI-TOF, API20E and WGS | A 3-year investigation of *Aeromonas* recovered from intestinal and extraintestinal infections to characterize, for the first time, the incidence in Germany. The authors indicated that the contribution of *A. encheleia* to the illness is doubtful, as this species, according to them has not previously been linked to human disease and was co-isolated with *A. bestiarum*. However, both species have been found at a low incidence in faeces of children with diarrhoea in Mexico (Belló-López et al., 2020) and *A. encheleia* was recovered from an ankle fracture (Janda and Abbott 1998) and was identified by Demarta et al. (2000) in association with gastroenteritis in young children in Italy. | Germany/ Schwartz et al., 2024 [17] |

Table S1. Cases involving the isolation of different *Aeromonas* strains or species in the same clinical sample. Continued 5

| ***Aeromonas* species (Nº of strains per patient or sample)** | **Origin** | **ID method** | **Comments** | **Country/ Reference** |
| --- | --- | --- | --- | --- |
| Five patients had 2 different species (NS), and one had 3 species (NS) | NS | NS | A 14-year retrospective study that investigated the clinical presentations of 105 patients with *Aeromonas* infections and analysed antimicrobial resistance in relation to disease progression and treatment. Most of the strains came from wounds and soft tissue infections (87.6%, 92 of 105 patients). | USA/Pineda-Reyes et al., 2024 [16] |
| *A. hydrophila* (1)  *A. caviae* (1) | Gastric aspirate from a deceased 4yo patient | MALDI-TOF and Phoenix M50 biochemical system | A foodborne outbreak was suspected to be associated to the unboiled stream water used to top up beef stew that was not properly re-cooked and that was considered to be the source of the infection that affected 72 people who developed gastrointestinal symptoms, and 3 died. The mixed infection identified in the gastric aspirate was the only confirmed case and involved *Aeromonas* strains that were similar to those found in the water stream, which tested positive for *Aeromonas hydrophila* by culture. | Uganda/ Nuwamanya et al., 2025 [18] |
| *A. caviae* (2)^e^ | Stool samples from 7 patients: 3yo male, 3yo female, 2yo male, 2 yo female, 20yo male, 58yo male (all without underlying diseases) and a 90yo female with comorbidity factors^f^ | MALDI-TOF, *rpoD* sequencing and genotyping with ERIC-PCR | *Ad hoc* study to determine the incidence of monomicrobial or polymicrobial infections involving different strains and/or species of *Aeromonas*, alone or in combination with other enteropathogens. The stool samples of 74 patients suffering from diarrhoea were investigated, genotyping 6 to 8 *Aeromonas* isolates from their faecal culture. Different strains were identified to the species level using the sequences of the *rpoD* gene.  More than one clone of the same or different *Aeromonas* species was found in 17 of 74 patients (23%). In 10 of these 17 cases, *Aeromonas* was the only pathogen. *A. caviae* was the most prevalent species, with two or three different strains identified in 9 patients, as shown in this table. In the remaining 7 cases, more than one strain of *A. caviae* (in most cases two strains) was isolated alongside with other enteropathogens: *Campylobacter jejuni* in 5 patients and *Salmonella enterica* in 2. | Spain/ Recio et al. (2026 submitted) |

^e^Each patient harbored a distinct strain pair; ^f^Hypertension, renal disease, and rheumatoid arthritis; NS: not specified

Table S1. Cases involving the isolation of different *Aeromonas* strains or species in the same clinical sample. Continued 6

| ***Aeromonas* species (Nº of strains per patient or sample)** | **Origin** | **ID method** | **Comments** | **Country/ Reference** |
| --- | --- | --- | --- | --- |
| *A. caviae* (3) | Stool from a 47yo male with no underlying diseases |  |  |  |
| *A. caviae* (2)  *A. hydrophila* (1) | Stool from an 88yo female with hypertension |  |  |  |
| *A. hydrophila* (1)  *A. rivipollensis* (1) | Stool sample from a 16yo male with no underlying diseases |  | . |  |
