# Supplementary material for: Gastrointestinal infection caused by five different strains of Aeromonas caviae and one of Aeromonas veronii: case report and review of the literature
Source: BMC Infect Dis. 2026 Apr 14;26:1016. doi: 10.1186/s12879-026-13287-6 (PMC13202911; doi:10.1186/s12879-026-13287-6)
Supplement: Supplementary file 3 — Supplementary Material 3 [file 12879_2026_13287_MOESM3_ESM.docx]

Table S2. Experimental studies that investigate the interactions of different *Aeromonas* strains or species in polymicrobial infections.

| ***Aeromonas* species (Nº of strains per patient or sample)** | **Origin** | **Animal Model or cell types used** | **Objective of the studies and main results** | **Country/ Reference** |
| --- | --- | --- | --- | --- |
| *A. sobria* (1)  *A. hydrophila* (1) | Aspirate obtained from a non-suppurative 10-mm-deep leg puncture wound sustained during diving in low-salinity water in a 19yo male diver patient presenting with decompression sickness | Rabbit ligated intestinal loop model and toxin assays on Y-1 adrenal cells | Both species were recovered in the initial culture upon admission, but only *A. hydrophila* was isolated in a subsequent culture performed 3 days later. Assays on adrenal cells showed that the *A. hydrophila* strain exhibited cytotoxicity and a cholera enterotoxin-like effect, whereas *A. sobria* did not show the latter. Rabbit ligated loop assays demonstrated that *A. sobria,* but not *A. hydrophila*, induces a fluid-accumulating response in the ileum. | USA/ Joseph et al., 1979 [8] |
| *A. hydrophila* (2) | Purulent discharge from a puncture wound in a 35yo male patient. Isolates from the previous study by Shak et al. (2011) with different susceptibility patterns (Table 3) | Septicaemic mouse model | Study designed to perform a comparative analysis of two wound isolates of *A. hydrophila* (E1 and E2) with environmental strains i.e., the type strain of *A. hydrophila* ATCC 7966T^a^ and of other species *A. aquariorum* AAK1^b^, *A. veronii* B565^c^, *A. salmonicida* A449^d^, and *A. caviae* Ae398^e^ to characterize genetic and functional differences that may explain the pathogenic potential of clinical isolates. E1, the most persistent strain in the infection, was more virulent than E2 and possessed several virulence factors (Act, T3SS, flagella, haemolysin, and a homolog of ExoA found in *Pseudomonas aeruginosa*) potentially related to its enhanced pathogenicity. | USA/ Grim et al., 2013 [92] |
| *A. hydrophila* (2) | Tissue cultures from the necrotizing fasciitis (NF) obtained immediately  after limb amputation | Septicaemic and intramuscular mouse models | Study conducted to compare the genomes of *A. hydrophila* strains causing NF with other clinical and environmental *Aeromonas* isolates to identify the genetic determinants contributing to their pathogenicity. In addition, the authors investigated the dissemination of bacteria in | USA/ Grim et al., 2014 [12] |

^a^GenBank accession no. CP000462.1; ^b^GenBank accession no. BAFL00000000.1; ^c^GenBank accession no. CP002607.1; ^d^GenBank BioProject PRJNA58631; ^e^GenBank accession no. WGS CACP00000000; Act: cytotoxic enterotoxin; T3SS: type 3 secretion system; ExoA: Exotoxin A.

Table S2. Experimental studies that investigate the interactions of different *Aeromonas* strains or species in polymicrobial infections. Continued 1

| ***Aeromonas* species (Nº of strains per patient or sample)** | **Origin** | **Animal Model or cell types used** | **Objective of the studies and main results** | **Country/ Reference** |
| --- | --- | --- | --- | --- |
|  | in a young immunocompetent patient, followed by successive cultures (Table 3) |  | NF mouse model. Of the two (NF1&NF2) wound genetically distinct isolates, NF1 (the first isolate) was more virulent in the septicaemic mouse model, whereas NF2 showed greater virulence in the intramuscular model. Isolate NF2 exhibited extensive dissemination from muscular tissue to visceral organs, whereas NF1 appeared to have a lower tendency to spread beyond the fascial tissue. |  |
| *A. media* ^f^ (1)  *A. veronii* (1) | Stool of a 5mo Spanish child that showed fever and comorbidity factors^g^ | *Caenorhabditis elegans* virulence model. The experiments determined the time to death required to kill 50% of the worms (TD50) | The study evaluates the pathogenic behaviour of the pairs of *Aeromonas* strains recovered from the same mixed infections (natural pairs) and of the combinations with other isolates from different cases (experimental pairs) of the same or of different species.  Two of the five natural pairs showed enhanced virulence when tested together: TD50 for the pair *A. media* 76c + *A. veronii* 77c was 3 days while for strain 76c was of 8 days and for strain 77c 8.5 days. The same occurred for the pair *A. hydrophila* BVH25a + *A. veronii* BVH25b that show a TD50 of 4.5 days, while for the individual strains was 6.5 for BVH25a and 8.7 for BVH25b. In conclusion the infections with combined strains produce higher mortality than single-strain infections, so in many cases, the virulence of one *Aeromonas* strain increases in the presence of the other co-infecting strain. Experiments show that synergy occurred only between strains of different species. The most virulent strains were not those with the greatest number of virulence genes, highlighting that *Aeromonas* virulence is complex and cannot be explained solely by the presence or absence of a potential virulence gene. | France/ Mosser et al., 2015 [13] |
| *A. hydrophila* (1)  *A. veronii* (1) | Respiratory tract sample from a 62yo man with comorbidity factors^h^ |  |  |  |
| *A. rivipollensis* (1)  *A. veronii* (1) | Respiratory tract, further data NS. |  |  |  |
| *A. sanarelli* (1)  *A. veronii* (1) | Wound, further data NS |  |  |  |
| *A. caviae* (2) | Stool faces of a patient from Spain. Further data NS |  |  |  |

^f^The strain of *Aeromonas media* was later reclassified by Talagrand-Reboul et al. (2017) as *Aeromonas rivipollensis* (Marti & Balcázar, 2015); ^g^Hydrocephalus with a ventriculoperitoneal shunt, and a catheter-associated coinfection; ^h^Bronchiectasis undergoing corticosteroid therapy; NS: not specified

Table S2. Experimental studies that investigate the interactions of different *Aeromonas* strains or species in polymicrobial infections. Continued 2

| ***Aeromonas* species (Nº of strains per patient or sample)** | **Origin** | **Animal Model or cell types used** | **Objective of the studies and main results** | **Country/ Reference** |
| --- | --- | --- | --- | --- |
| *A. hydrophila* (2) | Wound NF isolates from the above-described study of Grim et al. (2014). | Septicaemic and intramuscular mouse models | This study investigates the interactions among *A. hydrophila* NF strains, in a mouse model, to determine how co-infection influences virulence, host immune response, and physiopathology of the disease, compared to single strain infections.  The authors commented that, although apparently this case of NF may be considered monomicrobial because a single species was involved, genomic analysis revealed a mixed infection with four clonally related strains (NF2–NF4) and a phylogenetic different strain (NF1). The clonal group NF2–NF4 encodes exotoxin A (ExoA-positive) and differs from NF1 that is ExoA-negative. *In vivo* mouse experiments showed a different disease course after mixed infection: NF2 was highly virulent alone, but its virulence was attenuated in the presence of NF1. Dissemination of NF1 in the mixed infection was facilitated by ExoA virulence protein secreted by NF2, indicating a synergy between both strains that facilitated tissue barrier disruption. | USA/ Ponnusamy et al., 2016 [93] |
| *A. hydrophila-NF1* (1)  *A. hydrophila-NF2* (1) | Wound NF isolates from the study of Grim et al. (2014) and mutant strains (Δ) for different genes. | Murine macrophage cell line RAW 264.7 and mouse peritonitis and necrotizing fasciitis models | This study (complemented the previous study) elucidated how T6SS and ExoA contribute to the pathogenesis of *A. hydrophila* NF strains, by comparing their roles in single-strain and mixed-strain models of peritonitis and necrotizing fasciitis, focusing on bacterial dissemination, inter-strain interactions, and host immune response.  Experiments showed that NF1 strain possessed a unique T6SS effector toxin TseC, which played an important role in the direct killing of strain NF2 both *in vitro* and *in vivo*, as well as in bacterial phagocytosis and intracellular survival. | Spain/ Fernández-Bravo et al. 2019 [94] |

Table S2. Experimental studies that investigate the interactions of different *Aeromonas* strains or species in polymicrobial infections. Continued 3

| ***Aeromonas* species (Nº of strains per patient or sample)** | **Origin** | **Animal Model or cell types used** | **Objective of the studies and main results** | **Country/ Reference** |
| --- | --- | --- | --- | --- |
| *A. veronii* (2) | Cultures from an infected breast reconstruction flap, obtained two days after leech therapy for venous congestion. | *Drosophila melanogaster in vivo* model | Genome analysis of the two *A. veronii* strains (LR-14-3 and LR-14-4) and assessment of their virulence using a *Drosophila* model. Genome analysis of both strains revealed that they contained numerous antibiotic resistance genes (ARGs). The authors highlight the potential role of leeches as vectors for bacteria carrying multiple ARGs due to antimicrobial selective pressure.  The results in *Drosophila* model indicated that both strains had comparable virulence when tested individually, whereas when combined produced an enhanced virulence. | France, Barraud et al., 2020 [15] |
| *A. rivipollensis* (1)  *A. veronii* (1) | Stool of a 5mo Spanish child with diarrhoea and fever from the study of Mosser et al. (2015). The patient had predisposing and comorbidity factors^g^. | *Drosophila melanogaster* *in vivo* model | This study aimed to develop a *Drosophila melanogaster* systemic infection model for opportunistic pathogens and to evaluate its ability to distinguish between synergistic and antagonistic interactions of different combinations of *Aeromonas* strain isolated from human polymicrobial infections. The *Drosophila melanogaster* model demonstrates that coinfection can substantially alter virulence compared with single-strain infections, producing either synergistic or antagonistic effects. For instance, the pair *A. hydrophila* 25a/*A. veronii* 25b demonstrated comparable virulence whether tested together or separately, indicating a neutral interaction. In contrast, the *A. rivipollensis* 76c/*A. veronii* 77c combination showed an antagonistic effect. | France/ Robert et al., 2023 [95] |
| *A. hydrophila* (1)  *A. veronii* (1) | Respiratory tract sample from a 62yo man with comorbidities^h^ from the study of Mosser et al. (2015). |  |  |  |

^g^Hydrocephalus with a ventriculoperitoneal shunt, and a catheter-associated coinfection; ^h^Bronchiectasis undergoing corticosteroid therapy; T6SS: type 6 secretion system
